# Characterization of the complete mitochondrial genome and phylogenetic analysis of *Cnaphalocrocis patnalis* (Bradley 1981) (Lepidoptera: Crambidae)

**DOI:** 10.1080/23802359.2022.2116949

**Published:** 2022-09-08

**Authors:** Jihong Tang, Xue Tang, Hui Lu, Nanfang Lin, Chunxi Cheng, Baoqian Lyu, Jinhua Li

**Affiliations:** a Key Laboratory of Integrated Pest Management on Tropical Crops, Ministry of Agriculture and Rural Affairs, Environment and Plant Protection Institute, Chinese Academy of Tropical Agricultural Sciences, Haikou, China; b Hainan Key Laboratory for Biosafety Monitoring and Molecular Breeding in Off-Season Reproduction Regions, Sanya Research Institute, Chinese Academy of Tropical Agriculture Sciences, Sanya, Hainan, China; c Provincial Key Laboratory for Agricultural Pest Management of Mountainous Regions, Institute of Entomology, Guizhou University, Guiyang, China; d School of Plant Protection, Hainan University, Haikou, China

**Keywords:** Crambidae, *Cnaphalocrocis patnalis*, mitochondrial genome, phylogenetic relationship

## Abstract

*Cnaphalocrocis patnalis* (Bradley 1981) is a major pest that threatens the safety of rice production in the world. In the present study, we determined the complete mitogenome of *C. patnalis*. This mitogenome was 15,305 bp in length (GenBank accession No. OL449028), which contained two ribosomal RNA genes, 22 transfer RNAs, 13 protein-coding genes (PCGs) and one non-coding AT-rich region with a length of 344 bp. All the 22 tRNA genes displayed a typical clover-leaf structure, except for *trnS1*. Twelve PCGs were initiated by ATN codons, and *COX1* started with TTG. All the PCGs used the typical stop codon ‘TAA’ and ‘TAG.’ Phylogenetic tree demonstrated that *C. patnalis* belongs to the family Crambidae.

The rice leaf folder, *Cnaphalocrocis patnalis* Bradley (Lepidoptera: Crambidae) is a worldwide pest that damages rice through rolling and feeding with the rice leaves (Barrion et al. 1991). The leaf folder has caused up to 57% of the expected crop yield loss (Karim and Dean 2000). To date, research on this pest has focused on biological characteristics and patterns of occurrence, while studies on its evolutionary history have not been reported. In this study, we sequenced and annotated the complete mitogenome of *C. patnalis.*

Here, we sequenced and annotated the complete mitogenomes of *C. patnalis*, and created the phylogenetic tree for taxonomy classification. Individuals of *C. patnalis* were collected from Yazhou District in Sanya (18°19′22″N, 109°10′52″E), Hainan, China. All insect handling and experimental procedures were approved by the Ethics Committee of Chinese Academy of Tropical Agricultural Sciences (Hainan, China). The specimens were stored at −80 °C in the Environment and Plant Protection Institute, Chinese Academy of Tropical Agricultural Sciences, Haikou, China (Jihong Tang, jihong_23@163.com), under the accession number IN07040201-0001-00025. Total genomic DNA was extracted from single sample by the CTAB method (Reineke et al. 1998). The mitochondrial genome sequence was generated using Illumina HiSeq X TEN Sequencing System with 150 bp paired-end reads. The sequence was assembled by the MITObim software (Hahn et al. 2013) and checked by Geneious Primer (http://www. geneious.com/). The annotation of the mitochondrial genome sequence was mainly compared with the existing mitochondrial genomes of related species, and the annotation results were confirmed by the MITOS web server (Bernt et al. 2013). The sequence was submitted to GenBank under the accession number OL449028.

The *C. patnalis* mitogenome was 15,305 bp in length consisting of 13 PCGs, 22 tRNAs, two rRNAs, and a non-coding AT-rich region with a length of 344 bp. The nucleotide composition of *C. patnalis* mitogenome was biased toward AT at 96.2%, and the total base composition was 41.3% A, 54.9% T, 0.6% G, and 3.2% C. Twenty-two genes were encoded on the majority (J) strand, while 15 genes were encoded on minority (N) strand. Twelve PCGs started with ATN, except that *COX1* was initiated with TTG. Eleven PCGs used the typical stop codon ‘TAA,’ *NAD2* and *NAD5* used ‘TAG.’ The length of tRNA genes ranges from 48 to 72 bp and all of them can be folded into the typical clover-leaf secondary structure, except for *trnS1*, which lack dihydrouridine (DHU) arm. The 16S rRNA was 1350 bp long with an AT content of 85.2%, while the 12S rRNA was 779 bp long with an AT content of 86.5%.

Phylogenetic relationship of *C. patnalis* based on the concatenated datasets of the 13 PCGs from 17 Pyraloidea species was carried out, using *Ahamus yunnanensis* (Hepialoidea: Hepialidae) as an outgroup. The analyses were performed by the maximum likelihood using PhyloSuite v1.2.2 with 5000 bootstrap replicates (Zhang et al. 2020). FigTree v1.4.3 (http://tree.bio.ed.ac.uk/software/figtree/) to visualize the phylogenetic tree. The phylogenetic tree (Figure 1) demonstrated *C. patnalis* belongs to the family Crambidae, which accord with the conventional classification. In conclusion, the results of this study provide an essential DNA molecular database for further evolutionary and molecular research on Crambidae.

**Figure 1.**
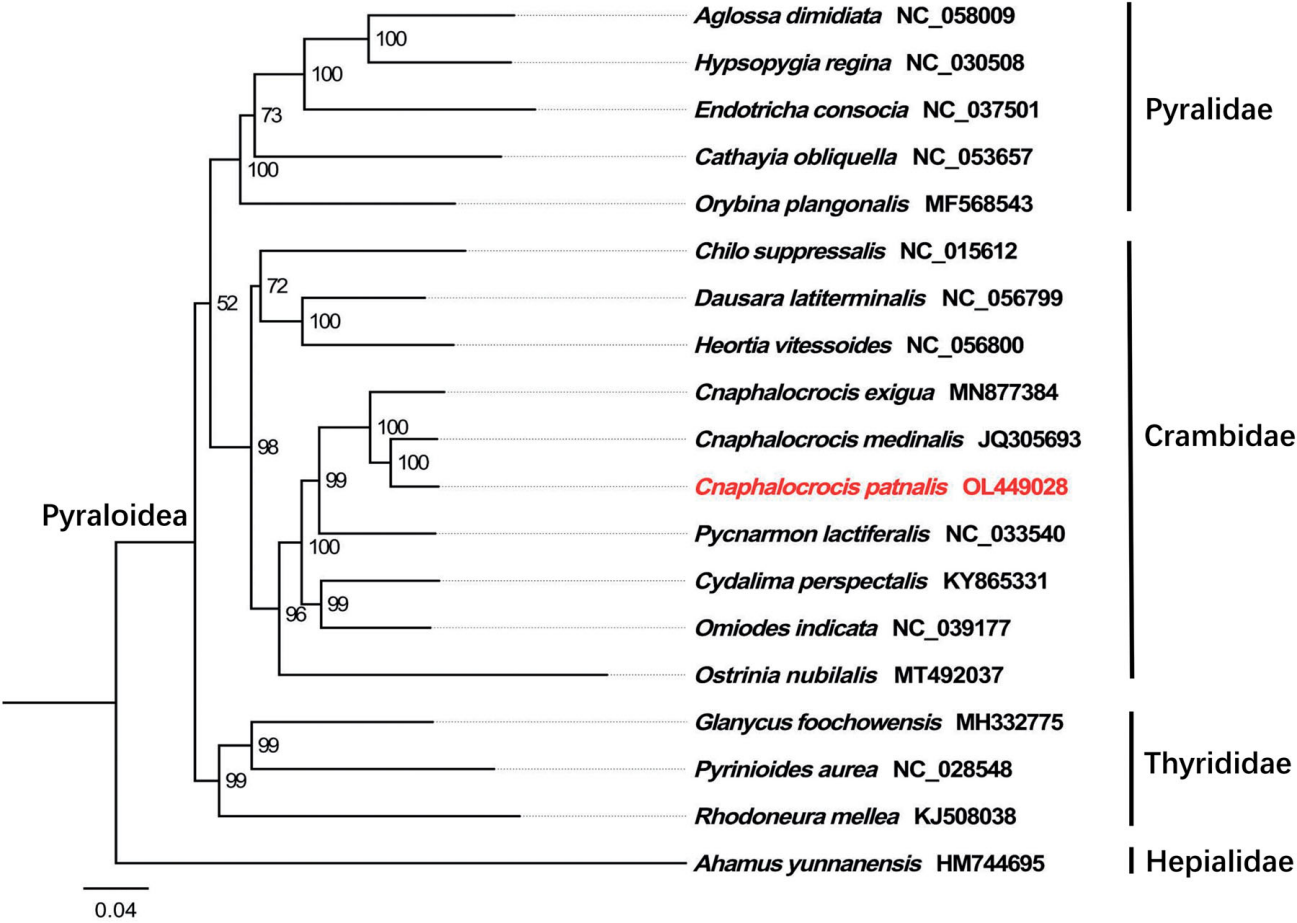
Phylogenetic tree showing the relationship between *Cnaphalocrocis patnalis* and 17 other species based on maximum likelihood method. GenBank accession numbers of each species were listed in the tree. Numbers on branches are bootstrap values. *Ahamus yunnanensis* was used as an outgroup.

## Data Availability

The genome sequence data that support the findings of this study are openly available in GenBank of NCBI at (https://www.ncbi.nlm.nih.gov/) under the accession No. OL449028. The associated BioProject, Bio-Sample and SRA numbers are PRJNA780076, SAMN23101374 and SRR16938303, respectively.
